# Effects of crude oil on survival and development in embryonated eggs in *Callinectes sapidus* Rathbun, 1896 (Decapoda, Portunidae)

**DOI:** 10.7717/peerj.5985

**Published:** 2018-12-11

**Authors:** Kelsie L. Kelly, Caz M. Taylor

**Affiliations:** 1 Brooklyn Law School, Brooklyn, NY, USA; 2 Department of Ecology and Evolutionary Biology, Tulane University, New Orleans, LA, USA

**Keywords:** Blue crab, *Callinectes sapidus*, Deepwater horizon, Oil spill, Blue crab development, Invertebrate embryos, Oil exposure, Prezoea

## Abstract

Blue crabs, *Callinectes sapidus* Rathbun, 1896, are ubiquitous along the Atlantic and Gulf coasts of the USA. These organisms play an integral role in the ecosystems of the Gulf of Mexico (GOM), where not only are they a keystone species, but are also socioeconomically important. The survival of embryonated eggs is necessary to ensure adequate recruitment into the next generation. Because the 2010 Deepwater Horizon oil spill (DWH) occurred during the peak of the blue crab spawning season, the incident likely impacted blue crab embryos. In order to assess the effect of oil on embryonic growth and development, we collected embryonated eggs from seven different female blue crabs from the GOM throughout the spawning season and exposed them to an oil concentration of 500 ppb (the approximate concentration of oil at the surface water near the site of the Deepwater Horizon oil rig). Exposure to oil at this concentration caused a significantly larger proportion of prezoeae vs. zoeae to hatch from embryonated eggs in experiments lasting longer than 4 days. Exposure to oil did not significantly affect overall survival or development rate. The prezoeal stage is a little-studied stage of blue crab development. Though it may or may not be a normal stage of development, this stage has been found to occur in suboptimal conditions and has lower survival than zoeal stages. The larger proportion of prezoeae following prolonged exposure to oil thus indicates that crude oil at concentrations likely to be experienced by crabs after the DWH spill negatively impacted the development of blue crab embryos. In addition to providing insight into the effects of the DWH, this study sheds light on embryonic development in blue crabs, a critical, but poorly investigated phase of this important species’ life cycle.

## Introduction

Marine organisms may be most vulnerable to the effects of toxicants at the embryonic stage due to the intense period of cellular activity that occurs during development (Connor, 1972; Lee et al., 1999). Studies examining the effects of various pollutants found detrimental effects on the growth and development of marine organisms (Lee & Oshima, 1998; Klumpp et al., 2002; Bellas et al., 2008). One pollutant to which marine organisms are likely to be exposed is crude oil released from natural seeps but also from oil spills, such as the Exxon Valdez spill in 1989 and the more recent Deepwater Horizon oil spill (DWH) in 2010. The DWH was the largest oil spill in US history and released approximately 4.1 million barrels of oil into the northern Gulf of Mexico (NGOM) from 20 April 2010 to 15 July 2010 (McNutt et al., 2012; Allan, Smith & Anderson, 2012). During the spill, oil concentrations in the surface waters were found to be as high as 500 ppb (Chiasson & Taylor, 2017; Wade et al., 2011). Previous research has shown that oil at concentrations as low as 0.4 ppb has significant impacts on the growth and development of herring embryos (*Clupea pallasi*) (Carls, Rice & Hose, 1999). Salmon embryos (*Oncorhynchus gorbuscha*) exposed to oil from the Exxon Valdez spill incurred genetic damage, which could be passed on to future offspring (Bue, Sharr & Seeb, 1998; Heintz et al., 2000). Heintz et al. (2000) found that polycyclic aromatic hydrocarbons, a class of over 100 compounds found in crude oil, at concentrations of 5.4 ppb resulted in a 15% decrease in juvenile survival. Sea urchin embryos (*Strongylocentrotus purpuratus*) that were exposed to crude oil experienced developmental delays, slower growth rate, abnormal cleavage and increased mortality (Allen, 1971).

One organism that may have been exposed to oil released from the DWH spill was the blue crab, *Callinectes sapidus* Rathbun, 1896. Blue crabs are highly abundant in the NGOM and are found in their juvenile and adult stages in near-shore estuarine benthic habitats (Guillory, Perry & VanderKooy, 2001). In the spring and summer, female blue crabs migrate offshore to spawn, often to barrier islands or sand shoals (Gelpi et al., 2009). The DWH overlapped with blue crab spawning in both timing and location (Gelpi et al., 2009; Grey et al., 2015). Female blue crabs carry eggs on their abdomen in a mass known as a ‘sponge’, and due to the primarily benthic lifestyle of blue crabs, prolonged exposure of the sponge to oiled sediments is likely (Burns & Teal, 1979; Hines, Lipcius & Haddon, 1987). In addition to exposure occurring in the year of the spill, exposure could occur for many years afterwards due to the persistence of elevated concentrations of oil within the sediments for up to 10 years (Burns & Teal, 1979).

It is important to understand the effect of oil on blue crabs due to the ecological and economic significance of this species within the Gulf of Mexico (GOM; Darnell et al., 2009; Gelpi et al., 2009; Alloy et al., 2015; Grey et al., 2015). Studies evaluating the effects of oil on blue crabs have focused on the larval and especially postlarval stages. Such studies have shown some sublethal effects, but have not demonstrated an increase in mortality or any reduction in population size as a result of exposure (Lee, Ryan & Neuhauser, 1976; Pearson et al., 1981; Wang & Stickle, 1988; Alloy et al., 2015; Giltz & Taylor, 2017; Chiasson & Taylor, 2017). However, because eggs may suffer prolonged exposure and because embryonic stages may be particularly vulnerable, it is necessary that we evaluate the effects of oil at the embryonic stage in order to investigate the potential damage caused by oil to the GOM blue crab population.

**Figure 1:**
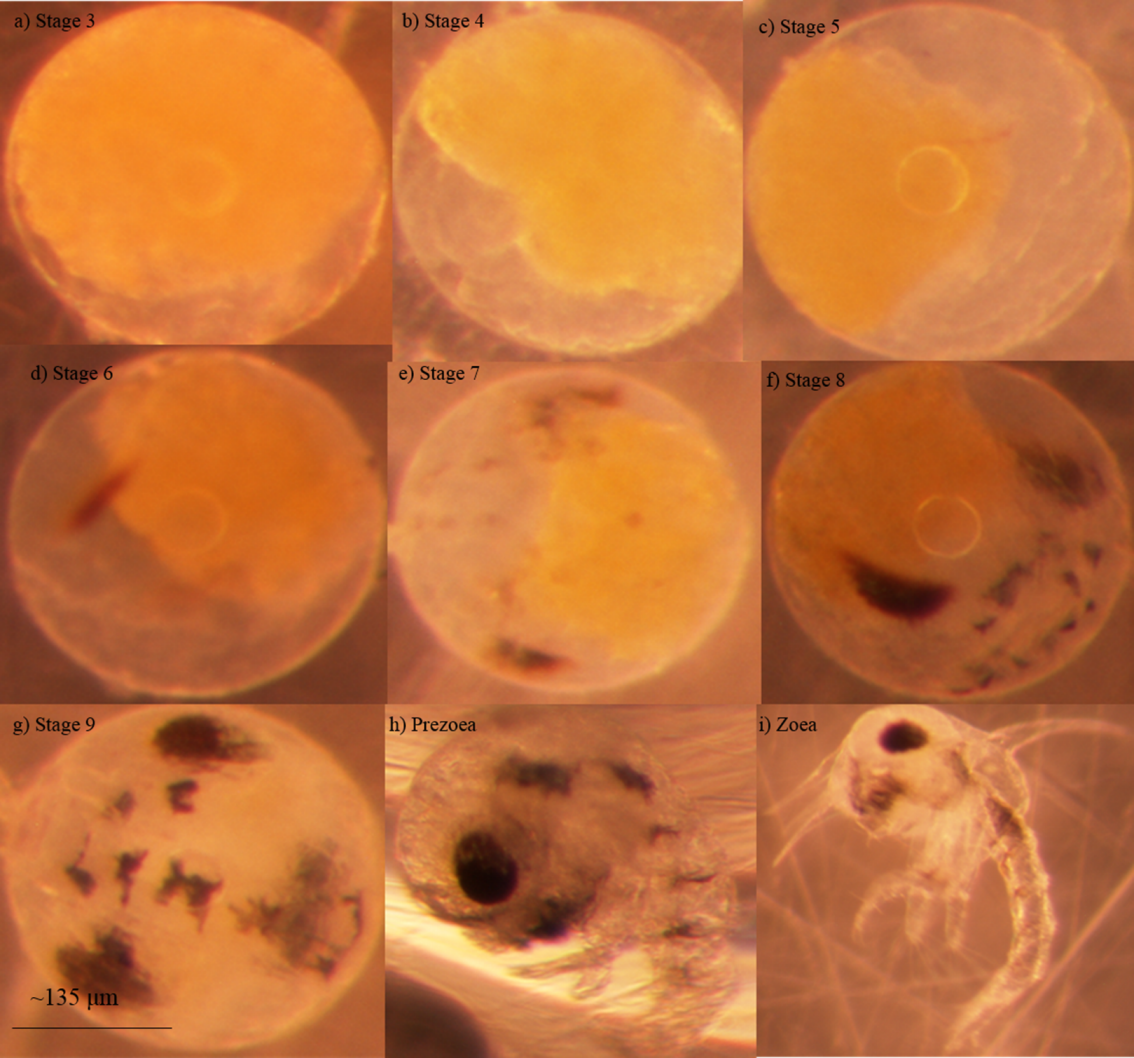
Developmental stages of embryonated eggs in *Callinectes sapidus* Embryonic stages of *Callinectes sapidus* Rathbun, 1896. (A) Stage 3 embryonated eggs are approximately ¾ yolk, (b) Stage 4 embryonated eggs are approximately ½ yolk, (C) Stage 5 embryonated eggs are approximately ¼ yolk, (D) Stage 6 embryonated eggs display faint eye spots, (E) Stage 7 embryonated eggs display faint abdominal lines, (F) Stage 8 embryonated eggs display darker and more defined abdominal lines, mouth parts are visible, and eyes are teardrop shaped, (G) Stage 9 embryonated eggs have distinct chromatophores, eyes are elliptical and dark, and heart beat is apparent in living specimens, (H) Larval prezoea, (I) Larval zoea. Average diameter for embryonated eggs is approximately 267 μm and average larval carapace width is approximately 278 μm (Darnell et al., 2009). Photographs by Kelsie Kelly.

Blue crab embryos undergo nine stages of development before hatching into a free-swimming larva known as a zoea (Fig. 1; DeVries, Epifanio & Dittel, 1983). Some researchers have noted an additional stage that seems to occur between the ninth embryonic stage and the zoeal stages known as a ‘prezoea’ (Robertson, 1938; Churchill, 1942). In the prezoeal stage, setae and spines are invaginated and the body is covered in a cuticle from which it must break free (Davis, 1965). There is some controversy as to whether the prezoeal stage is a natural, but brief, stage of development vs. an abnormality caused by poor environmental conditions (Sandoz & Rogers, 1944; Van Engel, 1958; Clark, Calazans & Pohle, 1998). Prezoeae are highly vulnerable due to a decreased swimming ability and have a reduced rate of survival such that a prolongation of this stage would have a negative impact on the organism (Clark, Calazans & Pohle, 1998).

In this study, we compared the development rates, survival and the stage upon hatching of embryonated blue crab eggs exposed to the concentration of oil at the site of the DWH to unexposed (control) embryonated eggs, in order to assess the effects of the crude oil on embryonic development.

## Materials and Methods

**Figure 2:**
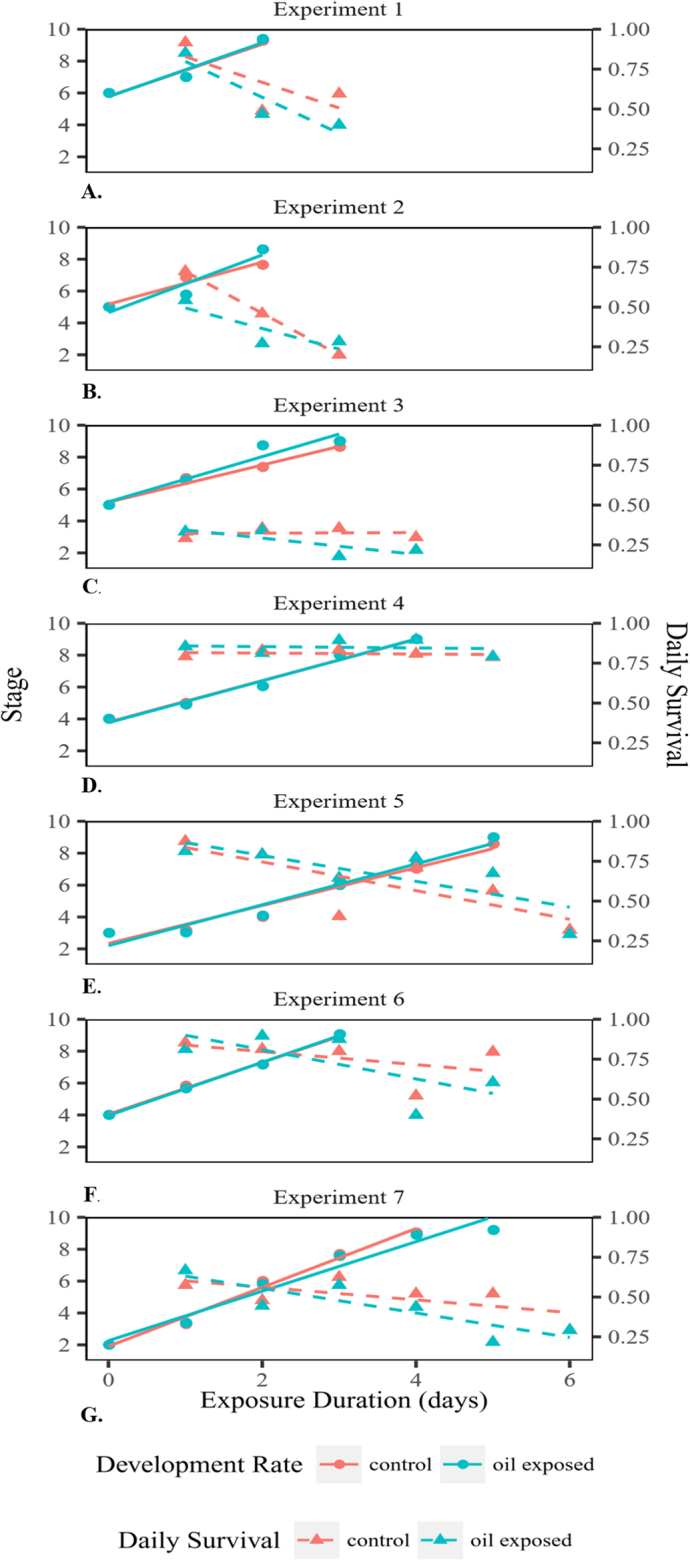
Developmental rate and survival of embryonated eggs and larvae. Developmental rate and survival of embryonated eggs and larvae over time exposed for each of the seven experiments (A–G).

We conducted an oil exposure experiment seven times on eggs collected from seven different female blue crabs. Egg masses were obtained from females, with permission from the Mississippi Department of Marine Resources, and were assigned an identification number 1–7 based on date caught. The crabs were collected via crab pots from within the Mississippi Sound (collection dates and locations for the seven egg masses were #1: 6 June 2015, 30°20′42″N 88°34′42″W; #2 and #3: 27 June 2015, 30°17′10″N 88°35′25″W; #4 and #5: 8 July 2015, 30°18′47″N 89°19′16″W; #6 and #7: 22 July 2015 30°18′47″N 89°17′68″W). For each experiment, the egg mass was removed from the female and the female was subsequently released. The egg mass was transported approximately one and a half hours away to Tulane University, New Orleans. As described by Lee et al. (1999), pieces of the egg mass were then placed in a container of seawater and shaken gently in order to dislodge the individual eggs from the egg mass. Eggs were taken up with a pipette and transferred individually into 48 wells of a 96-well plate with 99 μL of seawater with a salinity of 28 ppt (Lee, O’Malley & Oshima, 1996; Lee & Oshima, 1998; Lee et al., 1999). The eggs were then incubated at 28 °C for approximately 12 h and experimental trials commenced the following day. For each experiment, all eggs were derived from the egg mass of a single female. The majority of embryonated eggs in each egg mass were at the same initial developmental stage and all eggs selected were at the same (majority) stage. However, this initial stage varied among experiments. Water accommodated fractions (WAF) of South Louisiana Crude oil (MC252 surrogate) were prepared daily as described by Singer et al. (2000) for both oil-exposed and nonoil-exposed (control) eggs. The WAF was made with 28 ppt artificial seawater. A total of 150 mg of crude oil was added to 1.5 L water making the nominal crude oil concentration 100 ppm. The WAF was stirred for 24 h, after which it was diluted such that the ultimate concentration of oil within each oil-exposed well was 500 ppb (Chiasson & Taylor, 2017). Clean sea water was used in the control wells. Due to the limited information on the concentration of oil within the sediment at the site of the DWH, we used 500 ppb, the approximation for the highest oil concentration found at the surface water near the DWH after the spill (Chiasson & Taylor, 2017; Wade et al., 2011). This concentration provides a conservative estimate of the potential effects of the oil spill on the development of blue crab embryos. Full water changes were performed daily for both treatments with WAF readded to oil-exposed wells, so that the oil exposure was continuous for the duration of the experiment. Eggs were incubated at 28 °C in the dark until they hatched (Lee & Oshima, 1998; Lee et al., 1999). One 96-well plate from both the control and oil-exposed group was removed from the incubator daily, each egg in the plate was observed under a microscope, and then the removed plate was discarded from the experiment, because the changes in temperature and handling of eggs could interfere with development and alter results. Every egg in each daily removed plate was visually examined to determine its stage and whether it was alive (*n* = 48 per treatment per day). Once hatched, larvae were examined to see whether they were developmentally normal zoeae or prezoeae. Because the initial developmental stage (and therefore the time to hatch) varied among egg masses from different females, each experiment lasted a different number of days (Fig. 2).

Aliveness was determined by the colour and clarity of embryonated eggs. Living embryos were observed to have clear eggs with yellow yolk. Embryos in cloudy eggs with dark yolk that ranged in colour from dark yellow to orange were considered deceased. The stage of the embryo in each egg was determined by visually assessing distinct characteristics and morphological features (DeVries, Epifanio & Dittel, 1983; Fig. 1).

Once hatched, an individual was classified as either a developmentally normal zoea or a prezoea. A developmentally normal zoea had a heartbeat, lateral spines, a dorsal spine that was characteristically long and erect with a backwards arch, a telson, a rostrum, large eyes that were bilaterally symmetrical and fully pigmented, and was observed to swim freely and rapidly (Fig. 1I).

Prezoeae remained enveloped within a cuticle. While most prezoeae did have a heartbeat as well as large, fully pigmented and bilaterally symmetrical eyes, they did not display a visible rostrum or lateral spines. Prezoeae also had an impaired swimming ability. The dorsal spine of prezoeae was either not visible due to persistent invagination or it was noticeably shorter than the dorsal spine of a normal zoea (Fig. 1H). When visible, the shorter dorsal spine of some prezoeae presented a forward arch rather than the backward arch of the developmentally normal zoeae.

For each day of an experiment, we calculated the average stage of all embryonated eggs within the control and the oil-exposed groups and survival, which was the proportion that were alive in the removed well-plate. Each experiment was considered complete when greater than 90% of the eggs had either hatched or died in both treatment groups. For every plate within each experiment, we calculated the proportion of eggs that hatched and whether they hatched into zoeae vs. prezoeae.

The development rate for each experiment was calculated at the slope of the best fit regression line through average stage on each day. We assumed that development was linear and not affected by starting stage. A paired *t*-test was used to test whether the development rate was different in control vs. oil-exposed groups. An ANOVA was conducted to test whether the variation in daily survival was affected by female (ID number 1–7), treatment (oil-exposed vs. control), exposure time (number of days of exposure within experiment) or any interactions between them. We used ANOVA to test whether female, treatment, duration of experiment or any interaction explained the variation in the proportion of eggs that hatched as zoeae (vs. prezoeae).

## Results

Embryonated eggs developed at an average rate of 1.54 stages/day (Fig. 2). There was no significant difference in development rate between control (1.51 stages/day SD = 0.20) and oil-exposed groups (1.58 stages/day SD = 0.47; *t*(6) = −0.67, *p* = 0.53; Table 1; Fig. 2). The proportion that survived day to day decreased significantly with exposure time, but was not significantly affected by treatment or female ID (Fig. 2; Table 2A).

**Table 1:** Summary of experiments.

Experiment # (female id)	Initial stage (duration of experiment in days)	Development rate (stages/day):Control	Development rate (stages/day):Oil	Proportion of zoeae:Control	Proportion of zoeae:Oil	Proportion of prezoeae:Control	Proportion of prezoeae:Oil
1	6 (3)	1.70	1.81	1.0	0.79	0.0	0.21
2	5 (3)	1.84	2.53	0.80	0.82	0.20	0.18
3	5 (4)	1.23	1.26	0.58	0.88	0.42	0.12
4	4 (5)	1.45	1.40	0.97	0.73	0.03	0.27
5	3 (6)	1.51	1.55	0.93	0.64	0.07	0.36
6	4 (4)	1.40	1.22	0.93	0.35	0.07	0.65
7	2 (6)	1.42	1.30	0.93	0.31	0.03	0.69
Mean (SD)	4.29 (1.38)	1.51 (0.20)	1.58 (0.47)	0.88 (0.14)	0.65 (0.23)	0.12 (0.14)	0.35 (0.23)

**Note:**

Column 1, the experiment number (female identification number) of the female crab, from which all eggs were collected for the experiment; Column 2, the initial developmental stage of the eggs and the duration of the experiment in days; Column 3, the rate of development measured in the average number of stages progressed per day for the control group; Column 4, the rate of development measured in the average number of stages progressed per day for the oil-exposed group; Column 5, the proportion of zoeae out of the total number hatched in the control group; Column 6, the proportion of zoeae out of the total number hatched in the oil-exposed group; Column 7, the proportion of prezoeae out of the total number hatched in the control group; Column 8, the proportion of prezoeae out of the total number hatched in the oil-exposed group.

**Table 2:** ANOVA results.

	D*F*	Sum Sq	Mean Sq	*F*-Value	Pr(>*F*)
**(A)**					
Exposure time	1	0.2965	0.29648	6.084	0.0167*
Treatment	1	0.0113	0.01133	0.232	0.6316
Female id	1	0.1694	0.16943	3.477	0.0675
Exposure time:Treatment	1	0.0019	0.00192	0.039	0.8434
Exposure time:Female id	1	0.0258	0.02583	0.530	0.4696
Treatment:Female id	1	0.0053	0.00533	0.109	0.7420
Exposure time:Treatment:Female id	1	0.0213	0.02131	0.437	0.5112
Residuals	56	2.7292	0.04873		
**(B)**					
Duration of experiment	1	0.04041	0.04041	2.138	0.1940
Treatment	1	0.18784	0.18784	9.939	0.0197*
Female id	1	0.07116	0.07116	3.765	0.1004
Duration:Treatment	1	0.11603	0.11603	6.139	0.0480*
Duration:Female id	1	0.00004	0.00004	0.002	0.9631
Treatment:Female id	1	0.04576	0.04576	2.421	0.1707
Duration:Treatment:Female id	1	0.04399	0.04399	2.327	0.1780
Residuals	6	0.11340	0.01890		

**Notes:**

(A) Results of ANOVA testing how much the variation in proportion of embryos surviving in each well-plate on each day of each experiment was explained by female ID, treatment (oil-exposed vs. control) and exposure time within the experiment. Asterisk indicates statistical significance at the α = 0.05 level.

(B) Results of ANOVA testing how much the variation in proportion of embryos that hatched into zoeae was explained by female id, treatment (oil-exposed vs. control) and duration of experiment. Asterisk indicates statistical significance at the α = 0.05 level.

Prezoeae were observed in both the control and the oil-exposed treatment (Table 1). In five out of the seven experiments, a higher proportion of eggs hatched into prezoea in the oil-exposed group compared to the control treatment (Table 1).

**Figure 3:**
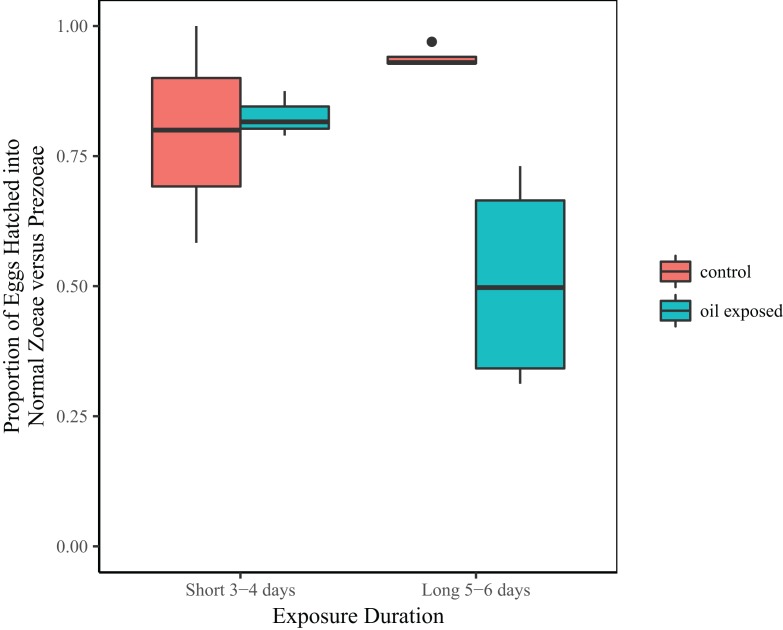
Proportion of developmentally normal zoeae out of total number of hatched larvae. Total proportion of embryonated eggs, which hatched into developmentally normal zoeae by treatment and time exposed.

Treatment and the interaction between treatment and duration were significant predictors of the proportion of zoeae vs. prezoeae hatched (Table 2B). In the shorter duration (3 and 4 day) experiments, there was no difference in proportion of eggs that hatched into zoeae between the oil-exposed and control eggs. However, in longer duration (5 and 6 day) experiments a significantly lower proportion of eggs hatched into zoeae vs. prezoeae in the oil-exposure treatments than in the controls (Fig. 3).

## Discussion

This study suggests that prolonged exposure to oil, even at low concentrations, can be detrimental to embryo development in blue crabs. Although no differences in survival or development rate were detected, we did see a significantly higher proportion of prezoeae in the oil-exposed eggs that hatched in experiments lasting longer than 4 days. Even if prezoeae were to be regarded as a normal stage of development, crabs only exist in this stage briefly and the increased number observed at this stage in the oil-exposed group when viewed once every 24 h indicates an increased duration of the prezoeal stage. Given the high mortality rate of decapod larvae during this stage, longer time spent as a prezoea would likely be detrimental (Clark, Calazans & Pohle, 1998). If prezoeae are an abnormality, an increase in prevalence is akin to an increase in mortality. Due to the restrictions of our experimental design, we were unable to establish whether the larger proportion of prezoea in longer oil-exposure experiments was due to the duration of the experiment or due to the exposure of embryos at an earlier stage. Future studies should focus on exposing embryonated eggs at earlier stages vs. later stages over varying amounts of time to distinguish between these potential causes.

Marine embryos are known to be effective biotic indicators and can be used to evaluate the overall health of an ecosystem (Klumpp & Von Westernhagen, 1995). Our study found a negative impact of oil on the life stage of one species, yet this could be indicative of a larger negative effect oil has had, and is having, on the ecological communities within the GOM. We suggest that our finding of a significantly higher proportion of prezoeae in oil-exposed treatments lasting longer than 4 days is evidence of a detrimental effect of oil, but further study is needed to better assess how this higher proportion of prezoeae might affect the population within the GOM.

Furthermore, because embryonated eggs were reared in an unnatural setting, our study does not allow us to tell whether or not prezoeae are a normal stage and would have molted into zoeae. Prezoeae as a normal developmental state of the blue crab would be consistent with the natural occurrence of prezoeae in other brachyuran crabs such as *Chasmagnathus granulatus* Dana, 1851 and *Chionoecetes bairdi* Rathbun, 1924, as well as in the more closely related species *Necora puber* Linnaeus, 1767 (Stone & Johnson, 1998; Greco et al., 2002; Lebour, 1928). Furthermore, Churchill (1942) and Robertson (1938) observed prezoeae during each of their individual assessments of the developmental stages of blue crabs.

While the findings in this experiment demonstrate a previously unknown impact of crude oil exposure on a novel system, they remain consistent with the conclusions of similar studies demonstrating the negative influence of oil on marine embryos (Fisher & Foss, 1993; Klumpp & Von Westernhagen, 1995; Hose & Brown, 1998). At best, prolonged oil exposure for lengthens the time spent in the vulnerable prezoeal stage and, at worst, triggers abnormal and fatal development.

## Supplemental Information

10.7717/peerj.5985/supp-1 Supplemental Information 1 Data for each embryonated egg evaluated for each of the 7 replicates for each day of the experiment. Metadata tab provides an explanation for each column used in the dataset.

## Additional Information and Declarations

### Data Availability

The following information was supplied regarding data availability: Data for each embryonated egg evaluated for each of the seven replicates for each day of the experiment. Metadata tab provides explanation for each column used in the dataset. The raw data has been supplied as a Supplementary File and at GRIIDC: Kelly, K. 2017. Data from effects of crude oil on embryonic development and early instars in Callinectes sapidus. UDI:R2.x214.000:0020. DOI: 10.7266/N7JW8BZ2.
